# Reference carbon cycle dataset for typical Chinese forests via colocated observations and data assimilation

**DOI:** 10.1038/s41597-021-00826-w

**Published:** 2021-02-02

**Authors:** Honglin He, Rong Ge, Xiaoli Ren, Li Zhang, Qingqing Chang, Qian Xu, Guoyi Zhou, Zongqiang Xie, Silong Wang, Huimin Wang, Qibin Zhang, Anzhi Wang, Zexin Fan, Yiping Zhang, Weijun Shen, Huajun Yin, Luxiang Lin, Mathew Williams, Guirui Yu

**Affiliations:** 1 Key Laboratory of Ecosystem Network Observation and Modeling, Institute of Geographic Sciences and Natural Resources Research, Chinese Academy of Sciences, Beijing, 100101 China; 2 National Ecosystem Science Data Center, Institute of Geographic Sciences and Natural Resources Research, Chinese Academy of Sciences, Beijing, 100101 China; 3 College of Resources and Environment, University of Chinese Academy of Sciences, Beijing, 100049 China; 4 University of Chinese Academy of Sciences, Beijing, 100049 China; 5 South China Botanical Garden, Chinese Academy of Sciences, Guangzhou, 510650 China; 6 State Key Laboratory of Vegetation and Environmental Change, Institute of Botany, Chinese Academy of Sciences, Beijing, 100093 China; 7 Institute of Applied Ecology, Chinese Academy of Sciences, Shenyang, 110016 China; 8 Key Laboratory of Tropical Forest Ecology, Xishuangbanna Tropical Botanical Garden, Chinese Academy of Sciences, Mengla, 666303 China; 9 Chengdu Institute of Biology, Chinese Academy of Sciences, Chengdu, 610041 China; 10 School of GeoSciences and National Centre for Earth Observation, University of Edinburgh, Edinburgh, EH9 3FF UK

**Keywords:** Ecosystem ecology, Ecological modelling, Forest ecology

## Abstract

Chinese forests cover most of the representative forest types in the Northern Hemisphere and function as a large carbon (C) sink in the global C cycle. The availability of long-term C dynamics observations is key to evaluating and understanding C sequestration of these forests. The Chinese Ecosystem Research Network has conducted normalized and systematic monitoring of the soil-biology-atmosphere-water cycle in Chinese forests since 2000. For the first time, a reference dataset of the decadal C cycle dynamics was produced for 10 typical Chinese forests after strict quality control, including biomass, leaf area index, litterfall, soil organic C, and the corresponding meteorological data. Based on these basic but time-discrete C-cycle elements, an assimilated dataset of key C cycle parameters and time-continuous C sequestration functions was generated via model-data fusion, including C allocation, turnover, and soil, vegetation, and ecosystem C storage. These reference data could be used as a benchmark for model development, evaluation and C cycle research under global climate change for typical forests in the Northern Hemisphere.

## Abstract (summary)

**Table:** 

Measurement(s)	photosynthetically active radiation • biomass • plant litter • leaf area index • soil organic carbon • CO2 flux • carbon cycling • temperature of air • rain • humidity of soil • humidity of air
Technology Type(s)	Sensor Device • forest inventory • litter trap • soil coring • eddy covariance • data assimilation
Factor Type(s)	plot
Sample Characteristic - Environment	forest biome
Sample Characteristic - Location	China

Machine-accessible metadata file describing the reported data: 10.6084/m9.figshare.13348154

## Background & Summary

Forests contain up to 80% of the terrestrial aboveground carbon (C) and 40% of the below-ground C and thus play a critical role in the terrestrial C cycle^1^. A recent study reveals that forests now serve as a net C sink for atmospheric CO_2_^2^. However, whether the forest C sink will persist under climate change remains largely uncertain^3,4^.Therefore, the availability of long-term and systematic observations of forest C dynamics is critical for improving the fundamental knowledge and understanding of forest C cycle processes and the robustness of forest C sink quantification and predictions.

The colocated network monitoring has developed over decades and provides a promising tool for obtaining long-term, intersite, multiple C cycle data^5,6^. Examples of such networks include the Long-Term Ecological Research Network (LTER), UK Environmental Research Network (ECN), and the Chinese Ecosystem Research Network (CERN). Among them, CERN has conducted systematic observations on the soil, atmosphere, biology, and water in accordance with unified monitoring standards since 2000^7,8^, and it has accumulated large amounts of long-term data on Chinese forests. The eastern China monsoon region, in particular, has been revealed to be one of the most significant C sink regions worldwide due to its special monsoonal climate, high nitrogen deposition, and relatively young age structure^9^. For this important forest, CERN can provide the only comprehensive dataset covering the typical forest types in this region in the Northern Hemisphere with few human activities, such as land use and cover change, destructive logging or sampling disturbances. This dataset can serve as an important benchmark for the analysis and assessment of regional and global C dynamics under global environmental change, such as climate change, increasing CO_2_ concentration and nitrogen deposition. In contrast to the numerous studies assessing aspects of the forest C cycle based on long-term and open-access data collected by ECN and LTER^10,11^, the integration and reanalysis of CERN data are still at the early stages. Recently, some researchers began to collect CERN data to investigate C cycle states and processes in forests, e.g., biomass and soil C density^12,13^, biodiversity^14^, tree mortality^15^, C allocation^16,17^, and ecosystem C turnover time^18,19^. These C cycle processes, e.g., C allocation and turnover, with various climate sensitivities collectively regulate the representation of how the forest C cycle responds to the climate^4,20,21^. At present, due to a lack of observations, even most state-of-the-art earth system models fail to accurately represent C allocation and turnover times^22,23^, which is largely responsible for the high uncertainty in the predictions of the forest C sink and its response to future climate change^24–26^. Therefore, it is critical and timely to 1) integrate and produce long-term, across-site, and systematic basic C cycle datasets based on CERN observations and 2) retrieve robust key C-cycle process parameters and time-continuous ecological function dataset (i.e., C sequestration) based on these basic reference data to better evaluate the spatiotemporal C dynamics of these important forests.

Here, we generated a time series and comprehensive dataset of the atmosphere, water, biological and soil C cycle based on CERN raw observations and statistical processing with strict collection criteria and quality control. On this basis, we also conducted a model-data fusion (MDF) framework to generate another assimilated dataset, including C cycle process parameters and C sequestration function products, neither of which can be obtained solely from observations. Moreover, the MDF framework achieved temporal interpolation from the basic time-discrete C cycle data to the time-continuous C sequestration function product. Recently, similar MDF-based time-continuous product was developed as novel benchmark in the International Land Model Benchmarking (ILAMB) project on C cycle^27,28^.

**Fig. 1:**
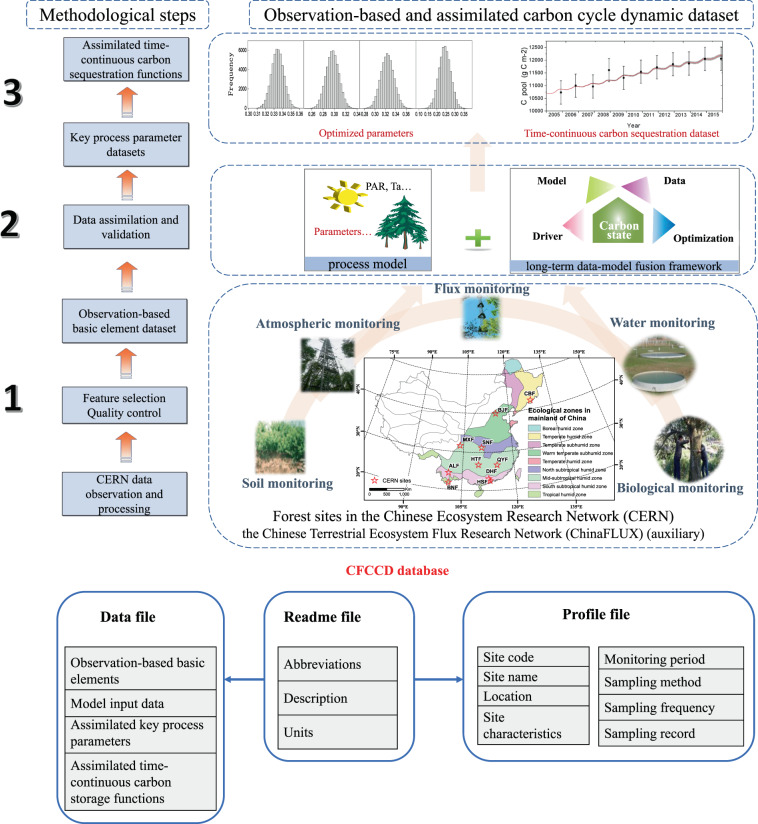
Schematic overview of the methodology and data products via the colocated CERN observations and long-term data assimilation. The flow diagram shows the methodological steps (left) and the data product systems (right) for the observation-based and assimilated databases. The bottom panel shows the general view of the data structure of the Chinese Forest Carbon Cycle Dynamics (CFCCD) database.

In this paper, we systematically described the estimation of basic C cycle elements and the MDF method to assimilate the C cycle parameters and sequestration functions of the Chinese Forest Carbon Cycle Dynamics (CFCCD) database (Fig. 1). This paper presents an observation-based basic dataset comprising monthly and annual atmospheric data, water data, and biological and soil C data from ten typical Chinese forests, an assimilated dataset of the C cycle parameters and time-continuous C sequestration (annual) from 2005 to 2015, and some flux data from the Chinese Terrestrial Ecosystem Flux Research Network (ChinaFLUX), which are used as auxiliary validation data in the MDF. This CFCCD database can provide as reference/benchmark for ecological modeling and C dynamics research under climate change for such typical forests in the Northern Hemisphere.

## Methods

We selected 10 permanent plots with long-term observations from CERN to include typical forest types of various ages in the East China monsoon forest region, including tropical rainforests, subtropical evergreen coniferous and broad-leaved mixed forests, warm temperate deciduous broad-leaved forests and temperate coniferous and broad-leaved forests, with evident precipitation and temperature gradients from south to north (Fig. 1). The spatial representativeness of the selected 10 sites across the Chinese forest region was evaluated by calculating the Euclidean distance based on various environmental factors. The 10 sites performed well and represented more than 80% of the Chinese forest region (Fig. S1). Of these forests, the Xishuangbanna tropical seasonal rainforest (BNF), Dinghu Mountain subtropical evergreen coniferous and broad-leaved mixed forest (DHF), Ailao Mountain subtropical evergreen broad-leaved forest (ALF), and Changbai Mountain temperate deciduous coniferous and broad-leaved mixed forest (CBF) are mature natural forests; the Shennongjia subtropical evergreen deciduous broad-leaved mixed forest (SNF) and Huitong subtropical evergreen broad-leaved forest (HTF) are natural secondary forests; and the other sites, i.e., the Beijing warm temperate deciduous broad-leaved mixed forest (BJF), Maoxian warm temperate deciduous coniferous mixed forest (MXF), Qianyanzhou subtropical evergreen artificial coniferous mixed forest (QYF), and Heshan subtropical evergreen broad-leaved forest (HSF), are plantations or middle- and young-age forests. All 10 sites are well protected and subject to minimal human activities, thus reflecting the C cycle dynamics under global environmental change, e.g., climate change, increasing CO_2_ and nitrogen deposition. The detailed characteristics of each plot can be found in their profiles in the CFCCD database.

There are three main steps to create the observation-based basic dataset and assimilated dataset of typical Chinese forests C cycle dynamics:

1. **Observation-based basic data acquisition**. An ensemble of daily atmospheric and water data at ten CERN sites were used as forcing datasets for MDF and future scientific analysis; biological and soil data were also collected from CERN and processed by quality control and statistical calculation as benchmark to constrain the model.
2. **Implementation of a multiple data-model fusion framework**. The Markov Chain Monte Carlo (MCMC) that integrated the Data Assimilation Linked Ecosystem Carbon (DALEC) model with multiple and dynamic observational data was used to retrieve C-cycle process parameters in a realistic disequilibrium state.
3. **Key process parameters and C function data assimilation**. The key parameters of the process-based C cycle model (DALEC) were determined via the model-data fusion method; then the ecosystem C sequestration datasets were simulated by forward running the DALEC model with optimized parameters and then validated based on observational data and other previous studies.

Each step is explained in more detail below.

### Observation-based basic data acquisition

#### Atmospheric and water data

In situ observations of daily air temperature (Ta), photosynthetically active radiation (PAR), relative humidity (RH), precipitation (Precip), and soil moisture (Sw) at the 10 sites from 2005 to 2015 were obtained from the CERN scientific and technological resources service system (http://www.cnern.org.cn/). These atmospheric and water data were mostly observed by an automatic meteorological station at each site. Among them, the PAR was estimated by a LI-COR LI-190SZ Quantum Sensor; Ta and RH were measured by a QMT110 sensor; Sw was estimated by a soil moisture neutron probe or the Time-Domain Reflectometry (TDR) soil moisture probe; the associated saturated soil water capacity (Sc) was measured by the cutting ring method to sample soil in each field campaign and the oven-drying method to measure saturated moisture content after the soil was soaked in water for 48 h; and Precip was artificially observed by CERN staff using an SM1-1 rain gauge. These monitoring data were collected in keeping with CERN’s protocols of observation and quality control^29,30^.

There were occasional missing data in time-continuous meteorological observations; therefore, the data were processed by standardized gap filling^31^. Specifically, for Ta, PAR, and RH, which were applied as model driver, we used a linear interpolation method to interpolate continuous missing data with less than three observations; otherwise, we established a regression model using the CERN observations and other observations from adjacent stations of the China Meteorology Administration (756 meteorological stations; http://data.cma.cn/en) to interpolate continuous missing data with more than three observations.

#### Biological data

##### Biomass.

At each site, the diameters at breast height (DBHs) and tree heights were measured for each tree in a regular inventory performed at least once every five years. The allometric equations of the DBH and/or tree heights with the biomasses of different plant tissues (i.e., leaves, branches, stems and roots) were developed at each site for various species based on the felled standard trees in the destructive plot. Then, we calculated the biomasses for the ten ecosystems using these allometric equations (FA02 table downloaded from http://www.cnern.org.cn/), which all passed the significance test (0.01 level) and have the R^2^ most above 0.9 when its estimation compare to observations from standard trees. For some unfelled species under protection, the allometric equations were obtained from Luo *et al*.^32^, which were developed based on national inventories and meta analyses from the published literature.

##### Litterfall.

The aboveground litterfall biomass was measured monthly by ten replicates with 1 m × 1 m baskets during the growing season or once during the nongrowing season. All collected litter was dried at 70 °C for 24 h in the laboratory and then weighed. To avoid the effects of wind on the measurement of litterfall biomass within an individual month, annual litterfall biomass data were finally adopted for each site.

##### LAI.

The leaf area index (LAI) at each site was measured optically with an LAI-2000 plant canopy analyzer (LI-COR, Lincoln, NE, USA) at least quarterly every year.

#### Soil data

Soil organic matter (SOM) was measured by the potassium dichromate oxidation titrimetric method. Soil bulk density (SBD) was measured by the cutting ring method in each field campaign at 10 forest sites. Soil particle size (i.e., soil mechanical composition) was measure by the laser particle analyzer. At least three samples were collected from each of the five soil layers (0–10, 10–20, 20–40, 40–60, and 60–100 cm) once every five years.

##### SOC.

The soil organic C (SOC) content was calculated from SOM, SBD, and volume percentage of gravel with particle size >2 mm at 10 forest sites as follows^33^:

1 $$SOC={\sum }_{i=1}^{n}0.58\times {H}_{i}\times {B}_{i}\times {O}_{i}\times (1-{\rm{\theta }})\times 100$$

where *SOC* is the soil organic C density (g C/m^2^) of all *n* layers, *H*_i_ is the soil thickness (cm), *B*_i_ is the soil bulk density (g/cm^3^), *O*_i_ is the SOM content of the *i*_th_ layer (%), and θ is the volume percentage (%) of gravel with particle size >2 mm. In the absence of soil bulk density or soil organic matter content measurements in some layers, the missing soil measurements corresponding to specific soil depths of theses forest ecosystems were supplemented according to the empirical formulas of the relationships between SOM/soil bulk density and soil depth in different layers, which were developed based on the long-term and across-site CERN soil observations^34^.

All these raw atmospheric, biological, and soil data mentioned above can be directly download from CERN scientific and technological resources service system (http://www.cnern.org.cn/data/initDRsearch) or obtained after online application via protocol sharing.

#### Auxiliary flux data

##### Net ecosystem exchange (NEE).

These data were obtained from ChinaFLUX (http://www.chinaflux.org/), covering CBF, QYF, and BNF. The data were aggregated to the daily time step from half-hourly CO_2_ flux data measured by the eddy covariance technique and processed with quality control and gap filling procedures^35^.

### Implementation of MDF method

**Fig. 2:**
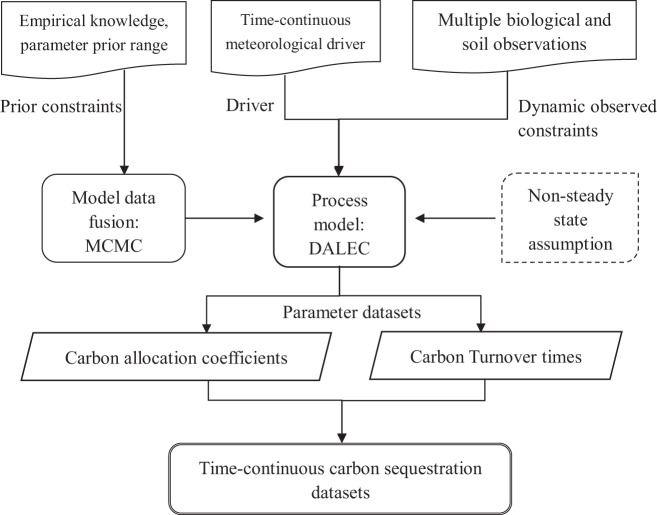
Flowchart of the generation of assimilated datasets in a multiple- and long-term data assimilation framework.

The assimilated data were retrieved from a multiple data-model fusion method (Fig. 2). Specifically, the long-term and dynamic observations of biomass, litterfall, LAI and SOC were used as the model constraint data; Ta, PAR, and RH were used as the meteorological driving data; and the metropolis simulated annealing algorithm, a variation in the MCMC technique^36,37^, was applied to retrieve the C cycle parameters (e.g., C allocation and C turnover times) against the observations and prior knowledge. Then, we forward-simulated the model to produce the dynamic and time-continuous changes in ecosystem C sequestration function.

Since the dynamic C cycle observations provided an effective solution to constrain the C cycle states without the steady state assumption (SSA), the novelty of our MDF framework involves estimating these C cycle dynamics in better agreement with the actual dynamic disequilibrium state^38^. Therefore, the uncertainty in allocation and turnover parameters and in C pool states have largely been reduced based on the time-series observations under the non-SSA (NSSA)^21,39,40^, thereby significantly enhancing the model’s ability to predict the C sequestration function^19,41,42^.

#### Carbon cycle process model description

DALEC is a box model of C pools connected via fluxes running at a daily time step and has been applied extensively to the MDF research^21,43^. Its main structure (i.e., C cycle, C allocation, and turnover process) is generally consistent with state-of-the-art process-based models (Fig. S2; Table S1), with five pools (i.e., foliage (Cf), fine root (Cr), woody (Cw, including branches, stems, and coarse roots), litter (Clit) and SOM (Csom)) for evergreen forests and an additional labile pool (Clab) of stored C that supports leaf flushing for deciduous forests. The C cycle was initiated with the canopy C influx: gross primary productivity (GPP), which was predicted by the Aggregated Canopy Model (ACM)^44^ (Appendix S1). After GPP is consumed by autotrophic respiration (Ra), the remaining photosynthate (NPP) is allocated to plant tissue pools (Cf, Cr, or Cw). The C exiting from all C reservoirs was based on a first order differential equation with various turnover rates, with temperature and moisture dependency on the turnover from the litter and soil pools. In contrast to the original DALEC model only with temperature scalar *f*_*Ta*_, here we added a new function *f*_*Sw*_ to express soil moisture pressure on litter and soil decomposition processes (Appendix S1). In general, the C pools and fluxes in DALEC were iteratively calculated at a daily time step and determined as a function of the key turnover and allocation parameters. A detailed model description can be found in Williams *et al*.^45^ and Fox *et al*.^46^.

#### Multiple data-model fusion at the nonsteady state

In a realistic disequilibrium state, C pools are time-variant, i.e., the C efflux is not equal to the C influx $\left(\frac{dC}{dt}\ne 0\right)$; thus, the MDF was run via the dynamic and long-term CERN observations to constrain the DALEC model at the non-steady state (Eq. 2). Here, to avoid the uncertainty arising from the spin-up process under SSA, we determined the initial state of the C pools by the initial observations of C stocks or by optimization (i.e., Clab, which cannot be directly observed). Then, the turnover and allocation parameters were retrieved under the disequilibrium state with dynamic environmental forcing. This method avoids the considerable uncertainties when invoking the SSA to estimate the initial state of C pools and the C cycle parameters(e.g., allocation coefficients and turnover rates)^39,40,47^, which could lead to obvious biases in C sequestration^19^.

2 $$\left\{\begin{array}{l}\Delta {C}_{i}\ne 0\\ {C}_{i}\left({\rm{t}}+1\right)={C}_{i}\left({\rm{t}}\right)+{I}_{i}\left({\rm{t}}\right)-{k}_{i}{C}_{i}\left({\rm{t}}\right),{\rm{i}}=1,2\ldots n\\ {C}_{i}\left({\rm{t}}=0\right)={C}_{i}0\end{array}\right.$$

where *C*_i_, *I*_i_, and *k*_i_ represent the size, input and turnover rate of the *i*_th_ C reservoir, respectively; *C*_i_0 represents the initial state of the *i*_th_ C reservoir; *t* represents the specific model-running time step (daily step); and Δ*C*_*i*_ represents the *i*_th_ C pool change between t day and t +1 day when applicable into actual calculation. According to the Bayesian theory, the posterior distributions of the parameters are calculated by maximizing the likelihood function (Eq. 3).

3 $$L={\prod }_{j=1}^{m}{\prod }_{i=1}^{{n}_{j}}\frac{1}{\sqrt{2\pi }{\sigma }_{j}}{e}^{-{\left({x}_{j,i}-{\mu }_{j,i}\left({\boldsymbol{P}}\right)\right)}^{2}/2{\sigma }_{j}^{2}}$$

where *L* is the integrated likelihood function; *m* is the number of multiple data types; *n* is the number of data points categorized by the *j*_th_ data type; *x*_j,i_ is the measured value composed of dynamic C cycle observations; *μ*_j,i_(*P*) represents the modeled fluxes and stocks based on parameters under the NSSA (*P*); and *σ*_j_ is the standard deviation of each data point classified by the *j*_th_ data type. Moreover, we imposed a sequence of ecological and dynamic constraints on the model parameter inter-relationships and pool dynamics (Appendix S2), which can significantly reduce uncertainty in model parameters and simulations^48^. The more detailed disequilibrium method can be found in our latest study^19^.

### Key C-cycle process parameters and C sequestration data assimilation

#### Key process parameter estimation

Here, we mainly focus on how the C input (i.e., the net primary productivity) partitioned into various plant pools (i.e., foliar, wood, and fine roots), i.e., allocation coefficients, which could be directly determined from the optimized parameters (Fig. S3) of the DALEC model after the step 2: MDF method. Another key process parameter, C turnover time, needs further simple statistical calculation based on the model simulations with optimized parameters. Turnover time is commonly estimated by the equation “τ = stock/flux”^20,49^. Since the C influx is not equal to the C efflux in the realistic dynamic disequilibrium state, the turnover time should be defined as the ratio between the mass of a C pool and its outgoing flux^50^. Note that with few natural and anthropogenic disturbances in these well-protected CERN sites^12,18^, the C efflux is approximately equivalent to the Rh from soil and litterfall (mortality) and Ra (growth) from vegetation. Hence, the turnover time for vegetation, soil, and whole ecosystem can be derived as follows:

4 $${\tau }_{veg}=\frac{{C}_{live}}{{I}_{live}-\Delta {C}_{live}}=\frac{{C}_{live}}{litterfall+{R}_{a}}$$

5 $${\tau }_{soil}=\frac{{C}_{dead}}{{I}_{dead}-\Delta {C}_{dead}}=\frac{{C}_{dead}}{{R}_{h}}$$

6 $${\tau }_{eco}=\frac{{C}_{eco}}{{I}_{eco}-\Delta {C}_{eco}}=\frac{{C}_{live+}{C}_{dead}}{{R}_{a}+{R}_{h}}$$

where *τ*_*veɡ*_, *τ*_*soil*_, and *τ*_eco_ refer to the biomass, soil and whole-ecosystem turnover times, respectively; *C*_live_, *C*_dead_ and *C*_eco_ refer to the live biomass C pool size (*C*_*f*_, *C*_*r*_, and *C*_*w*_,), dead organic C pool size (*C*_soil_ and *C*_litter_), and the whole-ecosystem C pool size, respectively; *I*_live_, *I*_dead_ and *I*_eco_ refer to the C input into the live biomass C pool, dead organic C pool, and whole ecosystem C pool, respectively; Δ*C*_live_, Δ*C*_*dead*_ and Δ*C*_eco_ refer to the changes in the live biomass C pool, dead organic C pool size, and whole-ecosystem C pool size, respectively; and *R*_*a*_, *R*_*h*_ and litterfall refer to the autotrophic and heterotrophic respiration, and turnover from all live C pools (i.e., foliage, fine root and woody pools),respectively, as calculated from the DALEC output driven with the estimated parameters during 2005–2015. Since the C reservoirs, fluxes, and turnover times are instantaneous values, we used the averages of the fluxes and reservoirs over multiple years to reflect the average turnover time during a specific period (i.e., 2005–2015).

#### Time-continuous C sequestration estimation

The optimized parameter values under the NSSA along with the initial observations of the corresponding C pool sizes were used in forward modeling driven by dynamic environmental variables from 2005 to 2015 to obtain the time-continuous soil and vegetation C storage^51^. The difference between the ecosystem C influx (GPP) and ecosystem respiration (Ra+Rh) is used to examine the ecosystem C sequestration, i.e., net ecosystem productivity (NEP). Similarly, the difference between the ecosystem C influx (GPP) and ecosystem autotrophic respiration (Ra) is used to examine the net primary ecosystem productivity (NPP).

## Data Records

The CFCCD database consists of three dataset types (Fig. 1) that were recorded in a series of Microsoft Excel files, which can be found on the Figshare repository at (10.6084/m9.figshare.12331400.v2)^52^. Among them, the ‘profile file’ (CFCCD Profile.xlsx) includes site and observation information, such as site code; site name; site plot area; site coordinates (longitude and latitude); site characteristics; site disturbance information; sampling method, frequency and sampling period associated with each atmospheric, water, biological, and soil variable. The ‘readme file’ (CFCCD Readme.xlsx) explains the abbreviations used in the ‘data file’ and ‘profile file’ and provides the units of all variables included. The ‘data file’ provides 3 datasets: (a) the observation-based basic element dataset, i.e., monthly/yearly observation-based basic C cycle elements with quality control and statistical calculation, including six atmospheric and water datasets as meteorological drivers (i.e., Ta, Precip, PAR, RH, Sw, and Sc), three biological C dynamic datasets (i.e., biomass; litterfall; and LAI), four soil C dynamic datasets (i.e., SOC, SOM, SBD, and soil texture), and one C flux dataset at some of the sites (NEE at CBF, QYF, and BNF); (b) the model input dataset, i.e., all the time-continuous meteorological drivers at model-running time step (daily step) used in model simulation and assimilation; (c) the assimilation dataset, including the assimilated parameter dataset based on the MDF method, i.e., allocation coefficients and turnover times retrieved specifically for each site; and the assimilated annual time-continuous ecosystem C sequestration functions consisting of vegetation C stock, soil C stock, and ecosystem productivity as well as respiration (Table 1).

**Table 1:** Element-parameter-function system for the data file in the Chinese Forest Carbon Cycle Dynamics (CFCCD) database.

Observation-based basic elements	Atmospheric element	air temperature (Ta)
		photosynthetically active radiation (PAR)
		relative humidity (RH)
	Water element	soil moisture (Sw)
		soil saturated moisture capacity (Sc)
		precipitation (Precip)
	Biological element	litterfall
		leaf area index (LAI)
		biomass for different plant tissues
	Soil element	soil organic carbon (SOC) density
		soil organic matter content (SOM)
		soil bulk density (SBD)
		soil texture (soil mechanical composition)
	Auxiliary carbon flux	NEE
Model input	Time-continuous meteorological drivers at model-running time step	
Assimilation dataset	Assimilated process parameters	carbon allocation coefficients
		vegetation, soil and ecosystem turnover times
	Assimilated C storage functions	soil, vegetation, and ecosystem carbon stocks
		ecosystem productivity, respiration and carbon sink

**Fig. 3:**
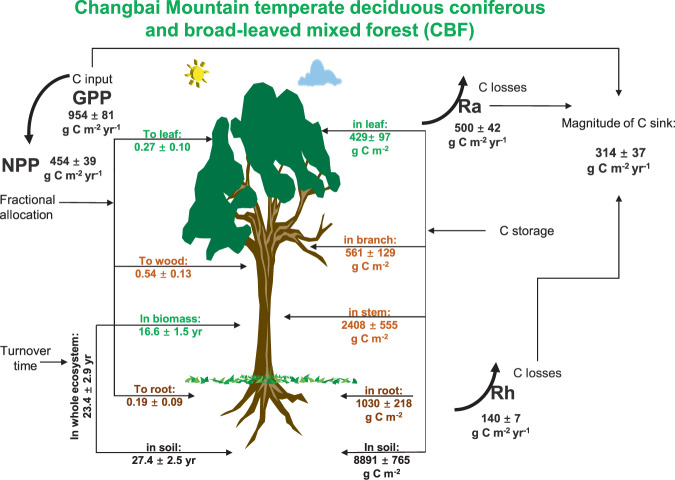
General map of how carbon flows in an ecosystem based on the Chinese Forest Carbon Cycle Dynamics (CFCCD) database, taking the Changbai Mountain temperate deciduous coniferous and broad-leaved mixed forest (CBF) as an example. The statistic values refer to the mean and the standard deviation of mean for various variables.

Specifically, the CFCCD ‘data folder’ includes records of 10 forest sites. The data time series at most of the sites cover the period from 2005 to the latest available year (2015), but those of the SNF, which were later incorporated into CERN, are from 2008 to 2015. The average C stock of the ten typical ecosystems is 21.6 kg C m^−2^. From north to south, with the increase in temperature and precipitation, the vegetation and soil stocks show a significant increase, indicating that the C stocks of the forest ecosystems in warmer and humid regions are higher than those in cold and dry regions (Table 2). Among the different C pools in the ecosystem, the soil C stock is the largest, accounting for 53.2% of the total C stock of the ecosystem; as the temperature and precipitation increase, the proportion of C stocks distributed in the soil gradually decreases, while the proportion of C stocks distributed in the vegetation gradually increases. In the past 10 years, all ten forest ecosystems function as C sinks, indicating a large C sequestration capacity in eastern China monsoon forests under climate change. Based the observation-based basic C cycle dataset, the optimized C cycle parameters, and the C sequestration function product, we can obtain a clear and transparent map showing how the C flows in different forest ecosystems (Fig. 3).

**Table 2:** Summary statistics (mean ± standard deviation of mean) for the assimilated dataset of biomass (Biomass_C), soil (SOC), ecosystem carbon stocks (Total_C), and net ecosystem productivity (NEP) during the 2005–2015 period at the ten forest sites.

Site_code	Biomass_C (g C m^−2^)	SOC (g C m^−2^)	Total_C (g C m^−2^)	NEP (g C m^−2^ yr^−1^)
CBF	4489 ± 618	8891 ± 765	13380 ± 1371	314 ± 37
BJF	5930 ± 552	4231 ± 329	10160 ± 879	266 ± 62
MXF	3214 ± 345	14795 ± 933	18009 ± 1269	371 ± 114
SNF	10381 ± 852	13377 ± 72	23758 ± 924	351 ± 37
HTF	12917 ± 525	7806 ± 324	20722 ± 848	231 ± 56
QYF	6542 ± 1233	6721 ± 572	13263 ± 1792	467 ± 118
ALF	24155 ± 612	29383 ± 452	53538 ± 1063	302 ± 65
DHF	13501 ± 894	9527 ± 268	23028 ± 631	232 ± 27
HSF	5339 ± 516	9068 ± 625	14407 ± 1138	301 ± 81
BNF	16141 ± 758	9896 ± 179	26037 ± 936	266 ± 72

## Technical Validation

### Observation-based validation

In terms of the observation-based dataset, the data at all CERN sites were obtained via internationally, widely used field samplers (e.g., rain gauge used for meteorological data), quantification methods (e.g., the allometric method used for biomass data), and laboratory analysis methods (e.g., the potassium dichromate oxidation titrimetric method used for SOM data). In addition, all measurements were subject to standard uniformity procedures from sampling to storage methods, which were undertaken by trained personnel at each CERN station. The CERN also presents detailed information on the protocols for standard observation, measurement, and laboratory analysis methods for the users to evaluate for themselves^29,30,53,54^.

Moreover, CERN has a three-level data quality control and validation system consisting of each station, subcenters (e.g., atmosphere, biology, soil, and water subcenters) and comprehensive center. To further improve the data quality, we also established a collaborative quality control framework among data users at the co-located network level and producers at the site level, focusing on the integrity, consistency, and reliability of the long-term, multisite and multielement observations during the production of the CFCCD database (Fig. S4). Specifically, we carried out data integrity analysis, consistency checks, and outlier elimination through time-series comparison, multisite comparison, multifactor comparison, and comparison with published literature, and then interpolated the missing data:

(1) Integrity check

Here, we mainly verified whether the observation frequency and sample information are complete, whether the metadata information of the data is missing, and preliminarily confirmed the degree of the missing data.

(2) Consistency check

The time consistency, spatial consistency, terminology consistency and element correlation of the observed data were systematically checked to determine, for example, whether the plant names were consistent in the inter-year community surveys, whether the names of the sample plots and the spatial sample areas were consistent in various sampling years, and whether the trend in the temporal variation in the elements followed relevant prior ecological knowledge.

(3) Detection of outliers

Statistical methods (such as the 3-σ criterion) are used to eliminate the abnormal values for the soil, biological, atmospheric and water elements. At the same time, the remaining existing observation values are compared with the results in the literature to validate and eliminate the abnormal values. Communication and confirmation are made with the staff at each station in terms of the input of the raw data, the calibration of the measuring instruments, the consistency of statistical calculation methods and the correction methods for the raw measurement.

Finally, after this strict quality control, the missing data were interpolated in accordance with different methods for different types of data, as described in detail in the Methods sections 2.1.1, 2.1.2, and 2.1.3.

### Assimilated dataset validation using in-situ measurements

**Fig. 4:**
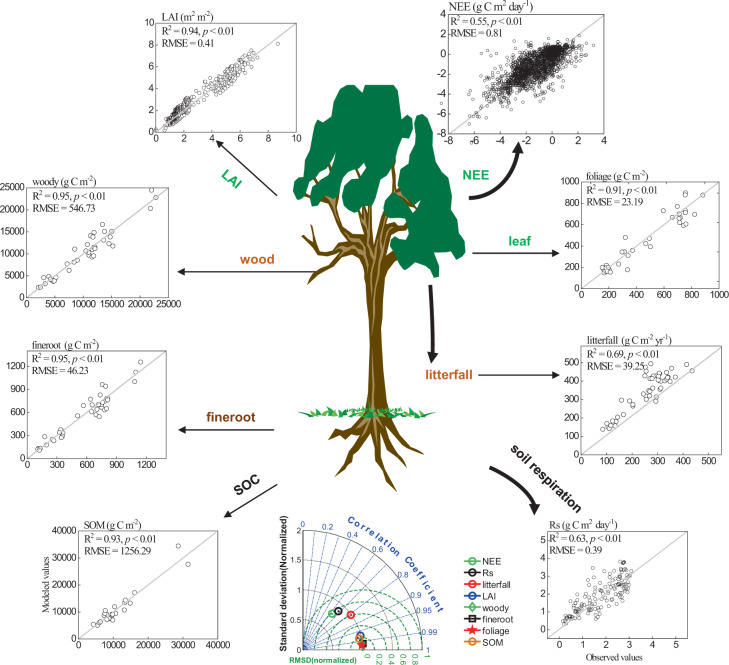
Comparisons between observed (in the x-axis) and modeled values (in the y-axis) at all sites under the data assimilation framework. The Taylor diagram (bottom panel) presents statistical tests (i.e., correlation coefficient, standard deviation, and root-mean-square deviation (RMSD)) for key fluxes and state variables as a summary of quality of fit.

For the assimilated dataset, we validated the performance by comparing the simulated vegetation and the soil C stocks and fluxes with the corresponding observations. The results showed good agreement, with the scatter points following the 1:1 line (Fig. 4). The taylor diagram showed that five stock-related variables had high correlation and low bias relative to observations. Specifically, the determination coefficients (*R*^2^) for the C stock-related variables varied between 0.91 and 0.95, and the root-mean-square errors (RMSEs) were small relative to their magnitudes. In addition to the biomass data, litterfall and SOC, we also added two datasets of C fluxes to validate the MDF performance, including the net ecosystem exchange (NEE) from ChinaFlUX, including CBF, QYF, and BNF, and soil respiration (Rs) data measured using static chamber-gas chromatography techniques at CBF, QYF, DHF, HSF, and BNF^55^. In contrast, the *R*^2^ values for C fluxes (NEE and Rs) were slightly lower (0.55−0.63), but the RMSEs were only 0.81 and 0.39 g C m^−2^ d^−1^, respectively, which fell well within the range of the C flux validation in MDF studies on eastern China forest ecosystems (e.g., Zhang *et al*.^18^). Moreover, we compared the directly observed pool-based increment (i.e., NEP = ΔBiomass + ΔSOC) to the modelled NEP via MDF; the result also showed a high consistency between the pool-based observation and model simulations based MDF (Fig. S5; *R*^2^ = 0.74, *p* < 0.01).

### Assimilated dataset validation using previous studies

**Fig. 5:**
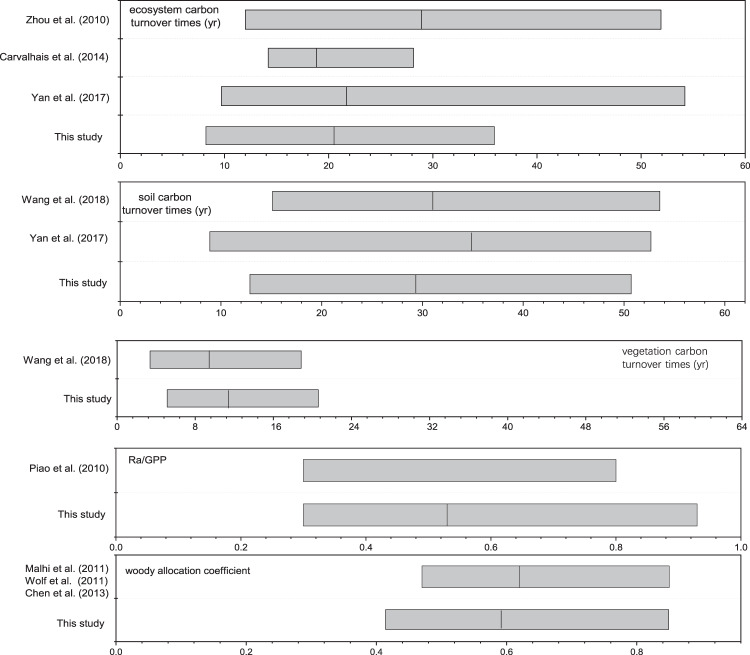
Comparison of assimilated parameters with those derived from other empirical studies.

Moreover, the observation-based constraint datasets and assimilated datasets were reviewed by international peers; several papers associated with this database have been published/submitted^13–15,18,19^. The optimized parameters (i.e., plant allocation, and the estimations of 𝜏_veg_, 𝜏_soil_, and 𝜏_eco_) under dynamic disequilibrium all showed high consistency with the existing empirical research based on field observations or experiments^20,25,56–61^ (Fig. 5). This indicated the reliability and robustness of our assimilated parameters under the realistic disequilibrium state.

### Uncertainties

Model-data fusion is a powerful method for the generation of improved simulation results via the combination of models with various data streams^62,63^. Model structure, model parameters, model assumption and observation data were the main sources of the uncertainties^63^.

The DALEC model is widely applied in various ecosystem across global scales with good performance^21^, and the model formulation bore similarities with the state-of-the-art process-based models^45,46^. Although we assumed that soils are a single homogeneous pool, which disregards the reality that soils consist of C that turnovers at different rates ranging from fractions of a year to centuries^64^, it has been challenging among earth system models to separate soils into different pools and quantify each pool’s turnover time due to lack of corresponded observed data^65^. DALEC assumes a single homogeneous soil pool thus to better assimilate with available observed information from CERN. Besides, our study mainly used the mean turnover time for whole vegetation or soil pool. Therefore, the single-soil-pool structure should not have significant on the estimation of mean soil turnover time and its further analysis. Besides, some forests are aggrading, and we conducted the MDF under the realistic dynamic disequilibrium assumption. Although the forest age was not directly considered in the model, the non-steady state estimation in this study based on long-term observational data (stock increments in aggrading forests) implicitly incorporated the age-structure-related effect on C cycle dynamics^19,42^, thus providing a proper estimation on carbon allocation or turnover process, as well as the C sequestration function.

We further conducted a sensitivity analysis to quantify the uncertainty sensitivity to input model parameters. The response variables for the sensitivity analysis are total annual NEE, GPP, Reco, and mean annual C pools. By modifying each parameter ± 10%, we calculated the percentage change in the response variables (VR) and the sensitivity index (β, ratio of the % change in response variable to % change in a parameter)^66^. Sensitivity analysis indicated that carbon fluxes and pools experienced similar sensitivity patterns (Table S2) considerably affected by parameters related to photosynthesis and C allocation. They were less affected by turnover as well as coefficient of correction. Since we collected field observations of LCMA, this measure was set as constant so that the model uncertainty over key photosynthesis parameters could be decreased, allowing emphasis on analyzing variation in allocation coefficients. Here the model allocation was well constrained by the time-series LAI and biomass of various plant tissues (Fig. S3), and thereby the allocation coefficients (especially allocation to wood and autotrophic respiration) showed high consistency with the empirical studies (Fig. 5).

The challenge of acquiring long-term and multiple observations covering different C cycle process is one of the inherent limitations in process-based model data fusion^40^. Here we mainly collected the biomass and SOC observations. Since CERN sites do not conduct the DOC fluxes observation, which is a small proportion of SOC^67^, the DOC fluxes were not produced in the model-data fusion analysis. This would bring uncertainty to carbon sink estimation in forests suffered soil erosion or land use change such as deforestation. Fortunately, the permanent plots at CERN sites are all protected well, but we still expect improved representations of carbon-water interaction process (e.g., DOC) into calibrated process-based models, to further help reduce the biases for the C balance of ecosystems regionally and globally.

Despite these inevitable uncertainties, the optimized key parameters and simulated C sequestration result are consistent with the site observational data and close to that of previous studies using different approaches (Figs. 4, 5 and S5). Overall, the CFCCD database provides high-quality open-access information on decadal C cycle dynamics in typical forests in China. The CFCCD database is the most comprehensive and up-to-date database covering decadal C cycle dynamics over the most representative forests in China and the Northern Hemisphere using measurement-based colocated networks; MDF-retrieved C cycle parameters, which are difficult to solely obtain from observations; and time-continuous C storage functions for long-term C cycle state evaluation under climate change. This reference dataset can be used to investigate the long-term trends in ecological C cycle dynamics, to identify the forest C sink distribution in soil and vegetation and their association with C-cycle process parameters, and to evaluate and improve the ability of C cycle process models as benchmarks. Such knowledge will have strong implications improving our ability to evaluate and understand forest C cycle responses to global change and will be important in the implementation of C sequestration and mitigation by policy makers.

## Supplementary information

Supplementary Information

## Data Availability

The DALEC model and the model-data fusion code used to generate the assimilated data products can be obtained through the GitHub repository at (https://github.com/ultradove/model-data-fusion). Further questions can be directed towards: Rong Ge (ge7218@163.com).
