# Supplementary material for: Reference carbon cycle dataset for typical Chinese forests via colocated observations and data assimilation
Source: Sci Data. 2021 Feb 2;8:42. doi: 10.1038/s41597-021-00826-w (PMC7854661; doi:10.1038/s41597-021-00826-w)
Supplement: Supplementary file 1 — Supplementary Information [file 41597_2021_826_MOESM1_ESM.docx]

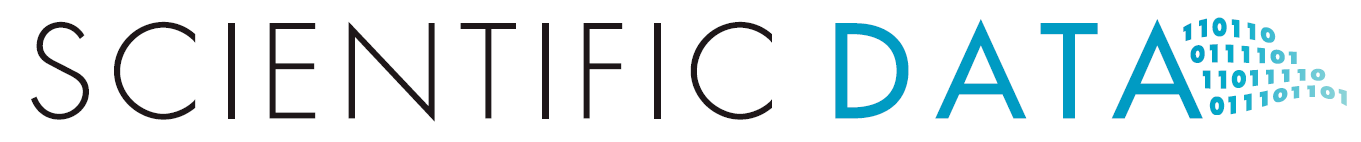


Supporting Information for

**Reference carbon cycle dataset for typical Chinese forests via colocated observations and data assimilation**

Honglin He^1,2,3†^, Rong Ge^1,2,4†^, Xiaoli Ren^1,2^, Li Zhang^1,2,3^, Qingqing Chang^1,2,4^, Qian Xu^1,2,4^, Guoyi Zhou^5^, Zongqiang Xie^6^, Silong Wang^7^, Huimin Wang^1^, Qibin Zhang^6^, Anzhi Wang^7^, Zexin Fan^8^, Yiping Zhang^8^, Weijun Shen^5^, Huajun Yin^9^, Luxiang Lin^8^, Mathew Williams^10^, Guirui Yu^1,2,3*^

1. Key Laboratory of Ecosystem Network Observation and Modeling, Institute of Geographic Sciences and Natural Resources Research, Chinese Academy of Sciences, Beijing, China, 2. National Ecosystem Science Data Center, Institute of Geographic Sciences and Natural Resources Research, Chinese Academy of Sciences, Beijing, 100101, China, 3. College of Resources and Environment, University of Chinese Academy of Sciences, Beijing, China, 4. University of Chinese Academy of Sciences, Beijing, China, 5. South China Botanical Garden, Chinese Academy of Sciences, Guangzhou, China, 6. State Key Laboratory of Vegetation and Environmental Change, Institute of Botany, Chinese Academy of Sciences, Beijing, China, 7. Institute of Applied Ecology, Chinese Academy of Sciences, Shenyang, China, 8. Key Laboratory of Tropical Forest Ecology, Xishuangbanna Tropical Botanical Garden, Chinese Academy of Sciences, Mengla, China, 9. Chengdu Institute of Biology, Chinese Academy of Sciences, Chengdu, China, 10. School of GeoSciences and National Centre for Earth Observation, University of Edinburgh, Edinburgh, UK

Table of Contents

[Supplementary Figures 3](#_Toc53486730)

[Figure S1. The spatial representativeness (a) and the relationship between mean annual temperature and precipitation (b) for the selected CERN sites across the Chinese forest region 3](#_Toc53486731)

[Figure S2. Structures of the Data Assimilation Linked Ecosystem Carbon (DALEC)-evergreen model (green) and the DALEC-deciduous model (green and blue) 4](#_Toc53486732)

[Figure S3. The optimized key parameters at ten sites aligned in a decreasing latitudinal gradient 5](#_Toc53486733)

[Figure S4. Framework of the collaborative quality control method between data users and producers 6](#_Toc53486734)

[Figure S5. NEP comparison between the direct, pool-based observation from biomass and SOC increment and the model simulation via model-data fusion 7](#_Toc53486735)

[Appendix 8](#_Toc53486730)

[Appendix S1: ACM and DALEC Model Description 8](#_Toc53486736)

[Appendix S2: Ecological Dynamic Constraints 10](#_Toc53486737)

[Supplementary Tables 12](#_Toc53486730)

[Table S1. Carbon allocation and turnover parameter descriptions in the Data Assimilation Linked Ecosystem Carbon (DALEC) 12](#_Toc53486738)

[Table S2. Sensitivities of carbon fluxes and pools to parameters in the DALEC model 13](#_Toc53486739)

[References 14](#_Toc53486740)


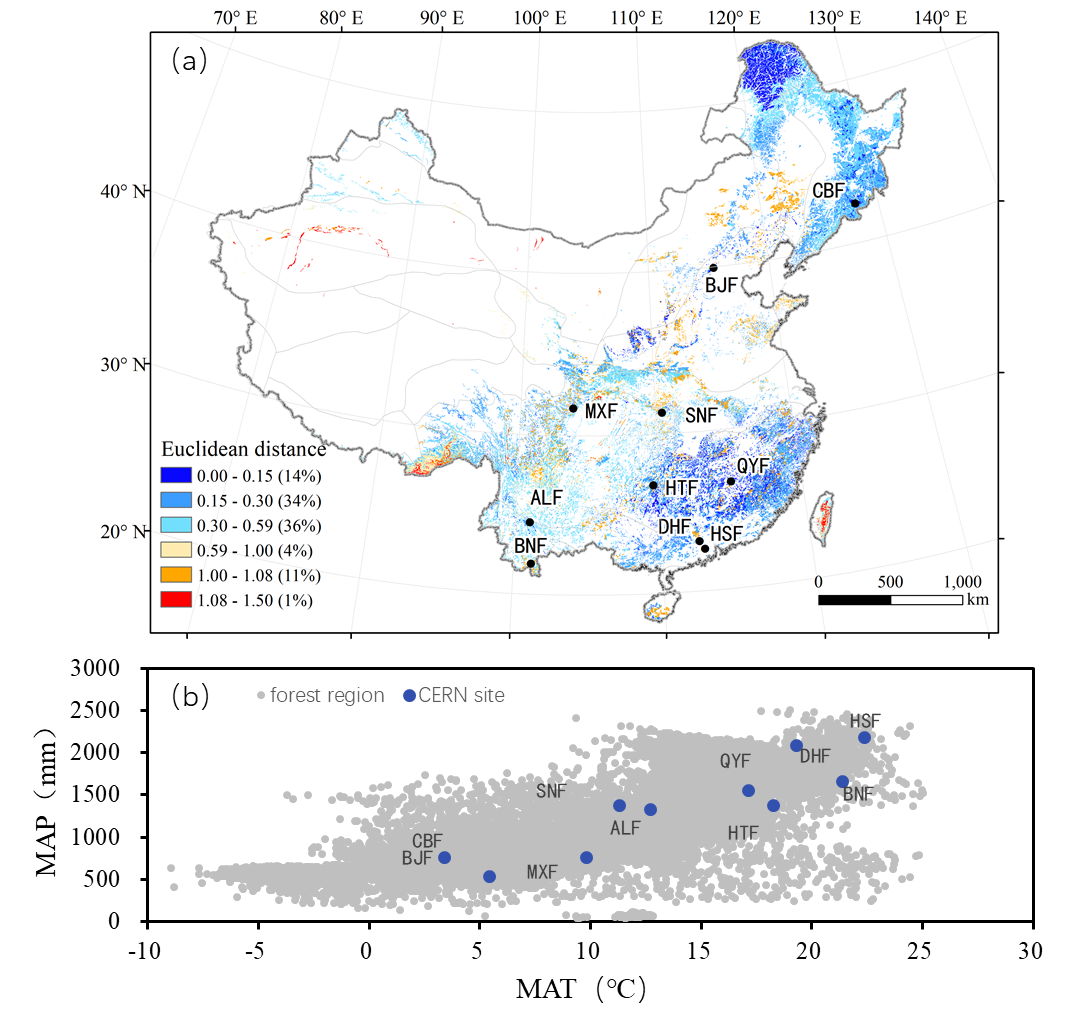


Figure S1. The spatial representativeness (a) and the relationship between mean annual temperature and precipitation (b) for the selected CERN sites across the Chinese forest region in the mainland of China. The representativeness of the 10 sites were estimated by combing the climatic factors (i.e., total solar radiation, air temperature, vapor pressure), soil properties (i.e., soil Ph and soil organic carbon content), topographic factor (i.e., altitude, slope and aspect) and the vegetation properties (i.e., enhanced vegetation index and vegetation type) to indicate the environmental characteristics of each 1-km pixel cell across Chinese forest region and CERN site. Next, the Euclidean distance from each pixel to the site was calculated to determine the representativeness of the selected CERN sites. The shorter distance means that the environment at the pixel was better represented by these selected CERN sites, and thus the higher representativeness of CERN sites. The results indicated that well represented areas (i.e., Euclidean distance < 0.59) covered more than 80% of the total areas


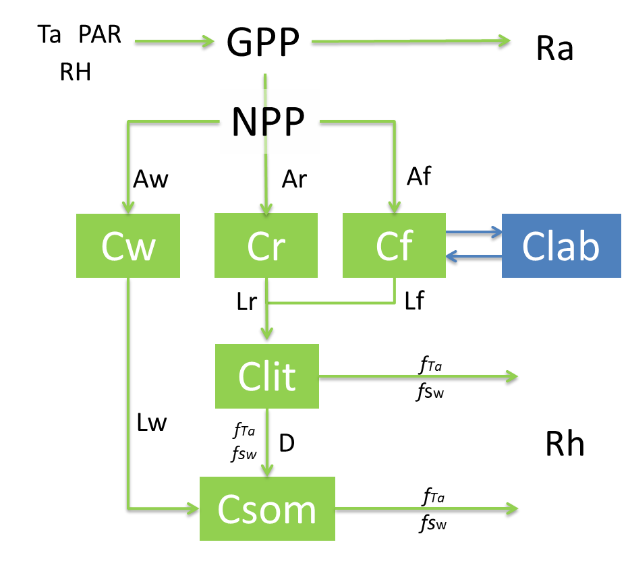


Figure S2. Structures of the Data Assimilation Linked Ecosystem Carbon (DALEC)-evergreen model (green) and the DALEC-deciduous model (green and blue), where GPP refers to the gross primary productivity; NPP refers to the net primary productivity, which is GPP remaining after consumption by the autotrophic respiration (Ra); Rh refers to the heterotrophic respiration; Aw, Ar, and Af refer to the carbon allocation coefficients to the woody, root, and foliage pools, respectively; Lw, Lr, Lf refer to the litterfall from the woody, root, and foliage pools, respectively; D refers to the Litter decomposition flux from litter pool to the soil organic matter pool; and *f*Ta and *f*sw refer to the temperature and moisture dependency on the turnover from the litter and soil pools.

Figure S3. The optimized key parameters at ten sites aligned in a decreasing latitudinal gradient. BNF: Xishuangbanna forest, HSF: Heshan forest, DHF: Dinghu mountain forest, ALF: Ailao mountain forest, QYF: Qianyanzhou forest, HTF: Huitong forest, SNF: Shennongjia forest, MXF: Maoxian forest, BJF: Beijing forest, CBF: Changbai mountain forest. Of them, BNF, DHF, ALF, and CBF are mature natural forests; SNF and HTF are natural secondary forests; and other sites, i.e., BJF, MXF, QYF, and HSF, are plantations or middle-aged and young forests.

Figure S4. Framework of the collaborative quality control method between data users and producers


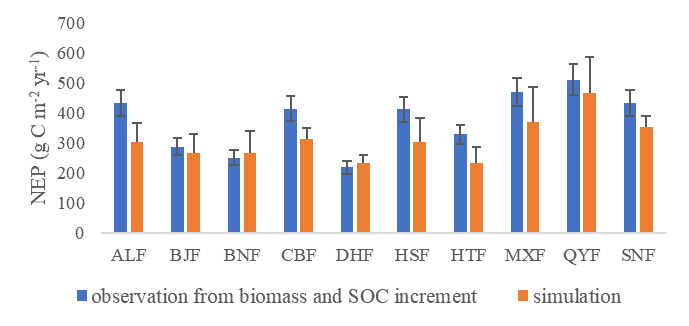


Figure S5. NEP comparison between the direct, pool-based observation from biomass and SOC increment and the model simulation via model-data fusion

Appendix S1: ACM and DALEC Model Description

The ACM is a big-leaf, daily time step model that estimates GPP as a function of LAI, foliar nitrogen, total daily irradiance, maximum and minimum daily temperature, day length, atmospheric CO_2_ concentration, soil–plant water potential, and total soil–plant hydraulic resistance.

$$GPP=\frac{E_{0}\times I\times g_{c}\times(C_{a}-C_{i})}{E_{0}\times I+g_{c}\times(C_{a}-C_{i})}\times(a_{2}\times s+a_{5})$$

where GPP is the gross primary productivity in g Cm^-2^ day^-1^, $E_{0}$ is the canopy level quantum yield (g C MJ^-1^ m^-2^ day^-1^), I is the irradiance (MJ m^-2^ day^-1^), $g_{c}$ is the canopy conductance (g C m^-2^ d^-1^), Ca is the atmospheric CO_2_ concentration (μmol mol^-1^), Ci is the CO_2_ concentration at site of carboxylation (µmol mol^-1^),$s$ is the day length (hrs), $a_{2}$ and $a_{5}$ are the daylength coefficient and daylength constant, respectively.

$$g_{c}=\frac{\left| \psi\right|^{a10}}{0.5T_{r}+a_{6}R_{tot}}$$

where $\psi$ is the maximum soil–leaf water potential difference (MPa), Tr is the daily temperature range (℃), Rtot is the total plant–soil hydraulic resistance (MPam^2^ s mmol^-1^), $a_{6}$ is the hydraulic coefficient, and a10 is the water potential constant.

$$C_{i}=\frac{1}{2}\left[ C_{a}+q-p \right]+\sqrt{\left( C_{a}+q-p \right)^{2}-4\left( C_{a}q-a_{3}p \right)}$$

$$p=\frac{a_{1}\times N\times L}{g_{c}}e^{a_{8}\times T_{max}}$$

$$q=a_{3}-a_{4}$$

where N is the average foliar N (gNm^-2^ leaf area), L is the LAI, estimated by L= Cf/LCMA, Tmax is the maximum daily temperature, a3 is the canopy CO_2_ compensation point, a4 is the canopy CO_2_ half saturation point, and a8 is the temperature coefficient.

$$E_{0}=\frac{a_{7}\times L^{2}}{L^{2}+a_{9}}$$

$$\sigma=-0.408\cos\left( \frac{2\pi t}{365} \right)$$

where $\sigma$ is the solar declination (radians), a7 is the maximum canopy quantum yield, and a9 is the LAI-canopy quantum yield coefficient.

ACM contains 10 parameters which have been calibrated using a fine-scale model (the Soil-Plant-Atmosphere model (SPA) (Williams et al., 1997; Fox et al., 2009) across a wide range of driving variables producing a ‘universal’ parameter set which maintains the essential behavior of the fine-scale model but at a much-reduced complexity. The detailed optimized value for ACM parameters were described below:

Parameter optimized value

a2 Daylength coefficient 0.0156

a3 Canopy CO2 compensation point 4.22

a4 Canopy CO2 half saturation point 208.9

a5 Daylength constant 0.0453

a6 Hydraulic coefficient 0.378

a7 Maximum canopy quantum yield 7.19

a8 Temperature coefficient 0.011

a9 LAI-canopy quantum yield coefficient 2.10

a10 Water potential constant 0.79

In contrast to the original DALEC version only considering temperature dependency, here we added a moisture scalar into the litter and soil decomposition process since Rh process is both temperature and moisture-sensitive. The widely adopted exponential response curve used here could improve the model structure to equally quantify the climatic sensitivity of turnover times to both temperature and moisture factors. The detailed equations are as follows:

$$R_{h\_litter}=C_{lit}\times\theta_{lit}\times f_{T}\times f_{w}$$

$$R_{h\_som}=C_{som}\times\theta_{som}\times f_{T}\times f_{w}$$

$$f_{W}={RH}^{{VPD}/\beta}$$

where the $R_{h\_litter}$ and $R_{h\_SOM}$ refer to the heterotrophic respiration from litter and soil organic matter (som) pool, respectively; $\theta_{lit}$ and $\theta_{som}$ refer to the baseline turnover rate of litter and som pool; $f_{T}$ and $f_{w}$ refer to the temperature and moisture scalar to adjust the real turnover rate, respectively; and RH is the relative humidity, VPD is saturation vapor pressure deficit, and β is a adjust constant.

Appendix S2: Ecological Dynamic Constraints

Ecological and dynamic constrains (EDCs) are the conditions we imposed on the inter-relationships between model parameters; therefore, the prior information of parameters not only consists of prescribed parameter ranges (Table 2), but also the ecological knowledge of actual conditions. Specifically, the EDCs include the turnover constraints and the root-foliar allocation parameter inter-relationships (used under both the SSA and NSSA), as well as the C pool growth dynamics (used only under the NSSA). These conditions refer to the constraints proposed by Bloom and Williams (2014) based on broad ecological knowledge of carbon dynamics to be the most ecologically suitable constraints for DALEC parameters and state variables, and are further determined according to the long-term observations and ecosystem characteristics in our research sites.

**1 Turnover Constraints**

We expect a faster litter turnover compared with soil organic matter (SOM) turnover (EDC 1), a faster conversion rate of litter to SOM compared with SOM turnover (EDC 2), and a faster ﬁne root turnover compared with SOM turnover (EDC 3). Therefore, we impose the following constraints on the relative relationship of turnover rates for different pools.

where *Ti* is the daily temperature values during an *N*-day time window (e.g., three years), is the temperature scalar to quantify responses of the carbon decay rate to changes in temperature, is the litter mineralization rate, is the turnover rate of soil organic carbon, and is the turnover rate of litter carbon.


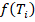

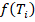

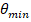

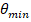

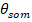

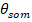

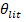

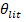


**2 Root-Foliar C Allocation Constraints**

Strong correlations are expected between foliar and ﬁne root carbon pools. We expect that the GPP allocated fraction to C_roo_ and C_fol_ (directly or via the labile C pool) are within a factor of 5 of each other (EDC 4). Meanwhile, the mean ﬁne root and foliar pool sizes are within a factor of 5 of each other (EDC 5). We constrain the C allocation and dynamics of the root and foliar pools:

where *C*_fol_ and *C*_roo_ are the mean foliar and ﬁne root carbon pool sizes over the model run period, respectively; and *f*_roo_, *f*f_ol_, and *f*_lab_ are the fractions of NPP allocated to roots, foliage, and labile, respectively.

**3 Carbon Pool Growth**

While we expect pools to potentially grow over time, we assume no recent disturbance and therefore limit the relative growth rate of pools. We constrain pool growth as follows:

where $\overline{C_{pool}^{year=1}}$ is the mean carbon pool size in year 1 and $\overline{C_{pool}^{year=n}}$ is the mean carbon pool size after *n*−1years. We choose a value of *G*_max_=0.1, which is equivalent to a 10% yearly growth rate (or doubling of carbon over 10 yr) as the maximum growth rate for each pool in EDC 6.

For ecosystems with no recent disturbance events, we propose that each pool is within an order of magnitude of its steady state attractor. We use mean gross primary productivity () as a proxy for long-term GPP to estimate the steady state attractor of four carbon pools (SOM, litter, wood and root). The steady state attractors for , , and are analytically derived as follows:


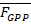

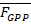

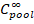

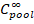

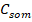

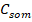

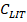

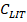

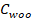

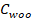

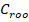

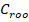

where *T* is the mean annual temperature (°C). For each pool, we impose an order-of-magnitude constraint on the proximity of from the initial value:


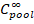

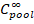

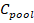

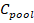

where $C_{pool}^{0}$ is the initial , , , and value for EDCs 7, 8, 9, and 10, respectively.


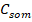

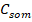

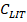

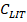

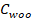

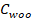

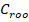

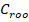


Table S1. Carbon allocation and turnover parameter descriptions in the Data Assimilation Linked Ecosystem Carbon (DALEC)

| parameter | description | range(low/high) |
| --- | --- | --- |
|  | **photosynthesis** |  |
| LCMA | Leaf C mass per leaf area (g C m-2) | 5-200 |
|  | **Turnover rates** |  |
| $\boldsymbol{\theta}_{\boldsymbol{min}}$ | decomposition rate (per day) | 1×10^-6^/0.01 |
| $\boldsymbol{\theta}_{\boldsymbol{fol}}$ | turnover rate of foliage | 1×10^-4^/0.1 |
| $\boldsymbol{\theta}_{\boldsymbol{woo}}$ | turnover rate of wood | 1×10^-6^/0.01 |
| $\boldsymbol{\theta}_{\boldsymbol{roo}}$ | turnover rate of roots | 1×10^-4^/0.01 |
| $\boldsymbol{\theta}_{\boldsymbol{lit}}$ | mineralization rate of litter | 1×10^-5^/0.1 |
| $\boldsymbol{\theta}_{\boldsymbol{som}}$ | mineralization rate of SOM/CWD | 1×10^-6^/0.01 |
|  | **Correction coefficients** |  |
| Rh_temp_ | Heterotrophic respiration exponential temperature dependence | 0.05/2 |
| β | Heterotrophic respiration exponential moisture dependence | 0.1/1.5 |
|  | **Allocation fractions** |  |
| $\boldsymbol{f}_{\boldsymbol{auto}}$ | fraction of GPP respired | 0.2/0.7 |
| *f*_fol_ | fraction of NPP allocated to foliage | 0.01/0.5 |
| *f*_roo_ | fraction of NPP2 allocated to roots | 0.01/0.5 |

Note: GPP is gross primary productivity; NPP is net primary productivity; NPP2 is NPP remaining after allocation to foliage. What remains after root allocation is allocated to wood. The prior range of each parameter refers to Fox *et al*. (2009) and Bloom *et al*. (2016). All the prior parameters wet set to be uniform distributions.

Table S2. Sensitivities of carbon fluxes and pools to parameters in the DALEC model

| Parameter | description | Function | $\beta$NEE | $\beta$RE | $\beta$GPP | $\beta$Cpool |
| --- | --- | --- | --- | --- | --- | --- |
| LCMA | Leaf C mass per area | photosynthesis | 29.99 | 5.04 | 5.80 | 0.13 |
| $\boldsymbol{f}_{\boldsymbol{auto}}$ | Fraction of GPP respired | C allocation | 27.05 | 3.68 | 4.40 | 0.10 |
| *f*_woo_ | Fraction of NPP allocated to wood | C allocation | 21.69 | 3.81 | 4.36 | 0.10 |
| *f*_fol_ | Fraction of NPP allocated to foliage | C allocation | 8.39 | 1.44 | 1.66 | 0.04 |
| *f*_roo_ | fraction of NPP2 allocated to root | C allocation | 6.29 | 1.10 | 1.26 | 0.03 |
| $\boldsymbol{\theta}_{\boldsymbol{fol}}$ | Turnover rate of foliage | turnover | 4.02 | 0.69 | 0.80 | 0.02 |
| $f_{W}(\beta)$ | Coefficient for moisture correction | coefficient of correction for turnover | 2.93 | 0.55 | 0.62 | 0.01 |
| $\boldsymbol{\theta}_{\boldsymbol{roo}}$ | Turnover rate of roots | turnover | 2.02 | 0.06 | 0.00 | 0.01 |
| $\boldsymbol{\theta}_{\boldsymbol{lit}}$ | Mineralization rate of fresh litter | turnover | 1.99 | 0.06 | 0.00 | 0.03 |
| $\boldsymbol{\theta}_{\boldsymbol{min}}$ | Decomposition rate | turnover | 1.72 | 0.05 | 0.00 | 0.03 |
| $\boldsymbol{\theta}_{\boldsymbol{som}}$ | Mineralization rate of SOM | turnover | 1.50 | 0.05 | 0.00 | 0.03 |
| $\boldsymbol{\theta}_{\boldsymbol{woo}}$ | Turnover rate of wood | turnover | 1.43 | 0.03 | 0.02 | 0.29 |
| $f_{T}(\mathrm{Rh}\mathrm{temp})$ | Coefficient for temperature correction | coefficient of correction for turnover | 0.78 | 0.02 | 0.00 | 0.02 |

References

Bloom, A. A. & Williams, M. Constraining ecosystem carbon dynamics in a data-limited world: integrating ecological "common sense" in a model–data fusion framework. *Biogeosciences* **12**, 1299-1315 (2014).

Fox, A. M. *et al.* The REFLEX project: Comparing different algorithms and implementations for the inversion of a terrestrial ecosystem model against eddy covariance data. *Agr Forest Meteorol* **149**, 1597-1615 (2009).

Williams, M. *et al.* Predicting gross primary productivity in terrestrial ecosystems. *Ecol Appl* **7**, 882-894 (1997).
